# Metabolic Plasticity in Dendritic Cell Responses: Implications in Allergic Asthma

**DOI:** 10.1155/2017/5134760

**Published:** 2017-12-14

**Authors:** Amarjit Mishra

**Affiliations:** Department of Pathobiology, College of Veterinary Medicine, 254 Greene Hall, Auburn University, Auburn, AL 36849, USA

## Abstract

Dendritic cells (DCs) are highly specialized in antigen presentation and play a pivotal role in the initiation, progression, and perpetuation of adaptive immune responses. Emerging immune pathways are being recognized increasingly for DCs and their subsets that differentially regulate T lymphocyte function based on the type and interactions with the antigen. However, these interactions not only alter the signaling process and DC function but also render metabolic plasticity. The current review focuses on the metabolic cues of DCs that coordinate DC activation and differentiation and discuss whether targeting these fundamental cellular processes have implications to control airway inflammation and adaptive immunity. Therefore, strategies using metabolism-based therapeutic manipulation of DC functions could be developed into novel treatments for airway inflammation and asthma.

## 1. Introduction

Dendritic cells (DCs) are heterogeneous population of rare hematopoietic cells that coevolved ontogenically with the emergence of the adaptive immune system during evolution. Myeloid-derived effector cells, such as DCs have a rapid turnover in general. For example, under normal steady-state conditions, the average residence time of airway epithelial DCs is only 3 days, suggesting that these cells are continuously made by the hematopoietic system [1]. DCs differentiate in the bone marrow (BM) from hematopoietic stem cells (HSCs), which give rise to a common dendritic progenitor (CDP) that is restricted to the DC fate based on their potential of FMS-like tyrosine kinase 3 (Flt3) expression and ability to respond to Flt3 ligand (Flt3L) for maintenance. The CDP produces preplasmacytoid DCs (pDCs) and preconventional DCs (cDCs), the latter of which leaves the BM and circulates in the blood before entering the lung tissue and further develops into DC subsets (*for review of DC subsets and nomenclature see* Merad et al.) [2–4]. The identification of distinct dendritic cell subset has fostered the concepts of distinct mechanism of tolerance and immunogenicity in adaptive immune response, which are triggered by pathogens, allergens, infections, and inflammation. Importantly, lung DCs are located mainly at the basolateral side of the epithelium and able to sense the surrounding tissues as well as the alveolar airspace to capture antigens and migrate to the lung-draining mediastinal lymph nodes, to maintain lung immune homeostasis in steady state and inflammation [5]. Under noninflammatory conditions, DCs remain in a quiescent resting state and are poorly immunogenic, whereas environmental triggers such as allergen, germline-encoded pattern recognition receptor (PRR) ligands, for example, Toll-like receptor (TLR) ligands, viruses, growth factors (granulocyte macrophage colony-stimulating factors (GM-CSF)), or inflammatory cytokines trigger the process of activation and maturation of DCs and make them highly immunogenic. In particular, the major lung CD11b^+^ cDC (conventional/classical dendritic cells (cDC2)) subset has recently been shown to initiate and effectively generate Th2-mediated adaptive immune responses to inhaled aeroallergens, such as house dust mite (HDM) [6–8]. Similar and overlapping functions of CD103^+^ lung DCs (cDC1) have been suggested to be associated with Th1 or Th2 responses in airway inflammation, whereas plasmacytoid DCs have poised towards type I interferon production, viral clearance, and tolerance [9–12]. In aggregate, monocyte-derived dendritic cells (moDCs; originally branched from common monocyte progenitors (cMoP) in the BM) maintained in the lung are capable to initiate Th2 immune responses in asthma [3, 7].

It is increasingly recognized that the activation and immune-priming function of DCs are coupled to profound alterations of the cellular metabolic state and are comprised of eliciting a DC-specific response. Moreover, evidence is accumulating for distinct metabolic requirements of these DC subsets for their immune-priming and immune-polarizing function that regulates airway inflammation. This metabolic plasticity of DC function in adaptive immunity could pave the way for our current understanding in asthma initiation, perpetuation, and progression. This review discusses how DC metabolism is interlinked with the adaptive immune pathways and whether these signaling cues are attractive targets for developing novel treatments for asthma and airway disease.

## 2. Metabolic Plasticity of the DC Progenitors

Dual metabolic regulation by long-term HSCs (LT-HSCs) during self-renewal and quiescence primarily relies on anaerobic glycolysis. Lineage-committed progenitors rapidly enter into the Krebs cycle to meet the unique set of bioenergetic demands required for cell proliferation and differentiation via the mitochondrial oxidative phosphorylation (OXPHOS) switch. Interestingly, several observations have identified that mitochondria in HSCs are relatively inactive and produce less mitochondrial reactive oxygen species (mit-ROS) as compared to the progenitor cells [13]. In the bone marrow, HSC entry into cell cycle is triggered by increased levels of ROS that corresponds to the metabolic switch from glycolysis to mitochondrial OXPHOS. This metabolic reprogramming provides the robust energy required for HSC differentiation into committed progenitors [13, 14]. Studies using the conditional inactivation of protein tyrosine phosphatase mitochondrial 1 (*Ptpmt1*) in HSCs show rapid inactivation of differentiation and cell divisions, which slows down the mitochondrial respiration and increases the anaerobic glycolysis. The substrates of *ptpmt1*, such as phosphatidylinositol phosphates (PIPs), activate the mitochondrial uncoupling protein 2 (UCP 2), thereby blocking the glucose-derived mitochondrial pyruvate oxidation in *Ptpmt1*-deficient HSCs and progenitors [15]. Mechanistically, the exit from quiescence of HSCs is sensed by low level of DNA damage or stress that is regulated by the master transducer of the DNA damage response signaling pathways, such as ATM (ataxia telangiectasia mutated). This, in turn, blocks the BH3-interacting domain death agonist BID (an effector of the ATM kinase in DNA damage response) to enter into differentiation [16]. Furthermore, mitochondrial carrier homolog 2 (MTCH2), an outer mitochondrial membrane receptor, acts as a negative regulator to increase mitochondrial metabolism and ROS productions, thereby determining the HSC fate [17, 18]. Collectively, these findings confirm the key drivers of HSC fate decision in which a low level of ATP through anaerobic glycolysis is required to prevent ROS generation and to maintain quiescence. In contrast, high ATP and ROS levels through fatty acid oxidation (FAO) pathway and tricarboxylic acid (TCA) cycle determine commitment and drive the differentiation through asymmetric cell divisions.

In the bone marrow, DC-committed progenitors (common dendritic cell progenitors or CDPs) originate from HSCs that give rise to preconventional DCs (pre-cDCs) and plasmacytoid DCs (pDCs) [19, 20]. The pre-cDCs further differentiated into cDC1 and cDC2 lineage and exit the BM and migrate to peripheral organs, such as the lung [21]. In contrast, the human monocyte-derived DCs (moDCs; equivalent to mouse Ly6C^hi^ moDCs) that originate from common monocyte progenitors (cMoP) are activated by LPS or CD40 ligand (CD40L) via peroxisome proliferator-activated receptor gamma (PPAR-*γ*) pathways for maturation and type 2 immune responses [22]. Increased expressions of the transcription factor PPAR-*γ* and PPAR-*γ* coactivator 1*α* (PGC1*α*) have also been implicated in the process of moDC differentiation through active lipid metabolism and mitochondrial biogenesis [23–25]. The ability of monocytes to differentiate into moDCs is primarily dependent on increased citrate synthase activity and the conversion of mitochondrial citrate to cytosolic acetyl-CoA, an important intermediate in the process of fatty acid synthesis [26]. This suggests that differentiations of DCs are integrated and regulated by mitochondrial functions and metabolic pathways of fatty acid biosynthesis. Analysis of mouse DC progenitors in the presence of the serine/threonine kinase, mammalian target of rapamycin complex 1 (mTORC1), impairs Flt3L-driven differentiation and mobilization of pDCs and cDCs from the bone marrow both *in vitro* and *in vivo* [27–29]. Consistent with mTOR function in DC development, a recent study by Wang et al. has identified tuberous sclerosis 1 (Tsc1) (a modulator of mTOR1 and mTOR2 activities), which acts as a negative regulator and blocks the differentiation of cDCs and pDCs from DC progenitors. This is further associated with increased expression of the transcription factor Myc, thereby is regulated in the biosynthetic and bioenergetic programs for DC development [30]. The expression of Myc is coupled by mTORC1 and is crucial for activation and expression of proteins involved in glycolytic pathway [31]. In particular, the Myc paralogue MYCL expression is increased in DC progenitors, indicating the broader importance of Myc in mitochondrial biogenesis and respiration [32, 33]. Altogether, these results address the fundamental bioenergetic properties of DC-committed progenitors and the regulatory pathways of DC differentiation. Further investigations in this area would highlight the metabolic framework of CDPs, pre-cDCs, and pre-pDCs in the context of their mobilization from the bone marrow into the bloodstream and tissue differentiation during steady state and inflammation.

## 3. Metabolic Sensors of DC Metabolism

The metabolic sensors on DCs translate the information of the cellular energy levels into the biological responses by different signaling modules. The important exogenous key metabolites that trigger the nutrient-sensitive anabolic and/or catabolic pathways to support DC differentiation and function are discussed here.

The recent identification of surface markers for both human and mouse DCs has enabled their purification with high efficiency. Currently, the state-of-the-art seahorse flux analyzer platform, developed by Agilent, is equipped to perform highly accurate real-time measurement of extracellular acidification (ECAR) and oxidative phosphorylation (OCR) as surrogate measures of cellular glycolysis and mitochondrial respiration, respectively. These fundamental bioenergetic measurements have enabled us to further study DC metabolism during steady-state and inflammatory condition in different tissues. The nutrient-sensitive PI3K-AKT-mTOR pathway promotes the production of ROS and regulates DC activation and proliferation by modulating glycolysis and anabolic metabolism. Compensatory to the glycolytic regulation, the Krebs/TCA cycle activity slows down and increases pentose phosphate pathway (PPP), thereby fuel nicotinamide adenine dinucleotide phosphate (NADPH) for the NADPH oxidase enzyme to generate ROS [34, 35]. Importantly, a regulatory role for the coenzyme *β*-nicotinamide adenine dinucleotide (NAD^+^) and its reduced form NADH in primary T cell development, proliferation, and differentiation have been recently reported [36–38]. However, similar roles for NAD^+^ and its reduced forms in DC immunometabolism are yet to be investigated. The activation of PI3K by growth factors and nutrients, such as glucose and amino acids, further increases the cellular levels of phosphatidylinositol-3, 4-biphosphate (PtdIns (4,5) P_2_) and PtdIns-3, 4, 5-triphosphate (PtdIns (3,4,5) P_3_). This induces a conformational change in AKT [39]. Studies using an inhibitor of mTOR, Tsc1-deficient cDCs (cDC2), indicate increased expression of the DC maturation markers CD80, CD40, and CD86 that identifies the essential role of the tumor suppressor Tsc 1 as a negative regulator of mTOR signaling for DC maturation [30]. PI3K-mTOR signaling pathway has essential roles in IFN-*α* production from pDCs and has been shown to selectively inhibit the transcriptional activation of IL6, TNF-*α*, and IL10 cytokines in the presence of the mTOR inhibitor rapamycin during TLR-mediated DC activation [40–43]. Another important key player in DC metabolism is hypoxia-inducible factor 1*α* (HIF1*α*), a transcription factor that acts as a sensor to low oxygen availability. HIF1*α* has been shown to be associated with TLR-dependent activation of DCs [44–46]. The prolyl hydroxylase 2 (PHD2) hydroxylates the two proresidues that are present in the O_2_-dependent degradation domain of the HIF1*α*, which primes HIF1*α* for degradation under normoxic conditions. In contrast, when O_2_ levels are low (hypoxic conditions), this hydroxylation is inhibited, which prevents the degradation of HIF1*α* and promotes the accumulation of HIF1*α* and HIF1*β* complexes on the promoters of glycolytic genes [47]. Notably, the LPS-induced IL-1*β* production and NLRP3 inflammasome activation in macrophages are mediated through metabolic reprogramming [48, 49]. Macrophage-derived mature IL-1*β* productions require two consecutive signals. Activation of HIF1*α* promotes the transcription of pro-IL-1*β* and is driven by accumulation of the TCA/Krebs cycle intermediate succinate [50], whereas the second signal is generated by multiple stimuli including ATP to activate NLRP3 inflammasomes, which is regulated via the glycolytic enzyme hexokinase [51]. This is important, since the macrophage-derived IL-1*β* production and NLRP3 activation have been shown to expand the IL-17-producing type 3 innate lymphoid cells (ILC3 cells) in obesity-associated airway hyperactivity [52]. Although HIF1*α* modulates the expression of several glycolytic enzymes and activates glycolysis, however, studies indicate that transient TLR-mediated DC activation does not rely on mTOR-HIF1*α* signaling. Unsurprisingly, in contrast to PI3K signaling, the early TLR-dependent DC activation is mediated by downstream AKT signaling nodes, which phosphorylates and activates the rate-limiting enzyme hexokinase 2 (HK2) by a TANK-binding kinase 1 (TBK1) and inhibitor of nuclear factor-*κ*B kinase subunit-*ε* (IKK *ε*) [53–55]. Collectively, these findings confirm that the TLR-dependent activation of DCs induces an early event of metabolic alterations. These bioenergetic demands are solely driven by glycolysis to further synthesize more fatty acids. Importantly, the metabolic alterations of DCs upon activation are supportive of a role for expansion of ER and Golgi to adopt secretory state, via an AKT-dependent signaling pathway. The cellular levels of AMP/ATP ratios determine the activation of the metabolic sensor AMP kinase (AMPK) and modulate the phosphorylation and activation, thereby inhibiting mTORC1 and antagonize the fatty acid synthesis pathways [56]. This in turn drives the catabolic processes, such as activating PGC1*α* and mitochondrial OXPHOS which are crucial in regulating DC activation [57, 58]. Several interesting observations regarding DC activation using AMPK-deficient DCs were made during these studies. First, AMPK knockdown increases TLR-induced glucose consumption and myeloid DC activation via CD40 signaling. Second, the effect of TLR-induced changes could be reversed by pharmacological activation of AMPK. Third, in the presence of resveratrol, activation of sirtuin1 (SIRT1) and PGC 1*α* suppresses HIF1*α*, which reduces mitochondrial membrane potential and ATP levels rendering DCs more tolerogenic [59, 60]. This is important since SIRT1 regulates the production of IL 27 and IFN-*β* through deacetylation of the transcription factor interferon regulatory factor 1 (IRF 1) and thereby regulates Th17-mediated immune pathways [61]. In aggregate, SIRT 1 function has been shown to promote the Th2-immune responses in airway inflammation by suppressing PPAR-*γ* activity in DCs [62]. Fourth, knockdown of the downstream partners of PGC 1*α*, for example, the transcriptional factor nuclear factor erythroid 2-related factor 2 (NRF2) or PPAR-*γ* in DCs enhances maturation, dysregulates redox homeostasis, and contributes to development and priming of CD4^+^ T cells [63–65]. These findings collectively support the concepts that AMPK-mediated signaling pathways are potentially linked to metabolic changes in DC activation and promote tolerogenicity via PGC 1*α* activation to facilitate catabolic pathways.

## 4. Metabolic Plasticity of DC Activation

Resting or immature DCs are characterized by their reduced cytokine secretory capacity, priming, and ability to activate T cells, which solely fuel from ATP productions by oxidative phosphorylation (OXPHOS) in mitochondria. These immature DCs express the germ-line encoded pattern-recognition receptors (PRRs), such as Toll-like receptors (TLRs), C-type lectin receptors (CLRs), that patrol and rapidly recognize and respond to the inflammatory triggers like environmental antigens, TLR ligands, and microbial products in the peripheral tissues under noninflammatory conditions [11]. It is noteworthy, although a “glycolytic burst” has long been demonstrated as a metabolic signature of TLR4 stimulation, more complex metabolic changes exist and are driven by whole microorganism and multiple TLR activation [66]. Therefore, the metabolic reprogramming of these cells will be of fundamental importance in the context of the tissue environment, nutrient availability, and disease state *in vivo* [67].

**Figure 1:**
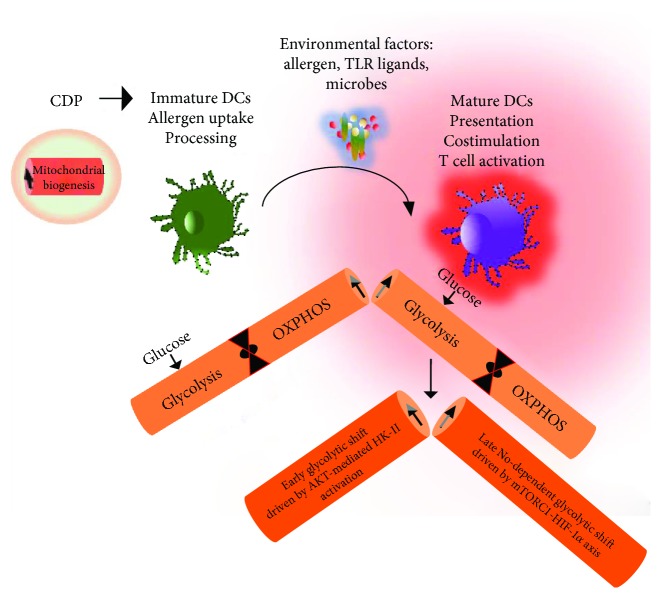
Basic preferences of DC metabolism. DCs originates from the common dendritic progenitors (CDPs) in the bone marrow (BM) that use mitochondrial oxidative phosphorylation (OXPHOS) as a key metabolic energy source and have increased mitochondrial biogenesis. These DC-committed progenitors egress the BM and circulate in the bloodstream and tissues as naïve immature DCs that promoted mitochondrial OXPHOS and shifted with the metabolic preferences upon allergen uptake and toll-like receptor (TLR) activation. The early glycolytic surge in activated DCs is primarily mediated via AKT pathways that phosphorylate and activate hexokinase II (the rate-limiting enzyme of the glycolytic pathway), whereas a late-occurring event of increased glycolysis is maintained by activated mTORC-HIF1*α* and is NO-dependent. NO: nitric oxide; mTORC1: mammalian target of rapamycin complex 1; HIF1*α*: hypoxia-inducible factor 1-alpha.

As summarized in Table 1, following encounter with the danger signals, DCs become activated, which are characterized by their enhanced ability to capture and process antigens and present the antigen-derived peptides to T cells. This in turn induces the genes encoding for cytokines, chemokines, and costimulatory molecules to exert T cell-specific immune responses. It has been recognized that homologous process of metabolic programming does exist in immature DCs as developing CD8^+^ T cells employ glucose to fuel the demand for fatty acid biosynthesis and glycolysis in their quiescent state to rapidly assemble and respond to restimulation by antigens [57, 68, 69]. However, several studies have identified that the metabolic reprogramming following TLR-induced activation of DCs and T cells differs strikingly. Intriguingly, the glycolytic surge in early activations of DCs does not employ ATP for additional necessary bioenergetic resources as compared to the T cells that primarily count on mitochondrial OXPHOS necessary for their activation [70, 71]. Rather crucial to this early rapid “glycolytic burst,” TLR activations in DCs are necessary for the de novo fatty acid biosynthesis via glucose-dependent citrate metabolism, which renders DCs with an immunogenic phenotype [44, 54, 72]. The early glycolytic changes are fundamental feature of activated moDCs, CD11b^+^ cDCs (cDC2), and CD8*α* ^+^ DCs (cDC1), and are primarily mediated via AKT-dependent signaling pathways. Furthermore, this contributes to the kinase-dependent activation of the rate-limiting glycolytic enzyme HK-II and its association with mitochondria [54]. Importantly, during inflammation, activation of DCs in response to TLR triggers is associated with an increased glycolysis that concomitantly shuts off mitochondrial OXPHOS. This glycolytic shift is both contributed from extracellular and intracellular glucose resources to meet the metabolic demands of DC immune activation and to support the synthesis of cytokine secretions [26, 73, 74]. Furthermore, activation-induced metabolic shift promotes the expression of inducible nitric oxide synthase (iNOS) and nitric oxide (NO) productions. This is mediated via PI (3) K signaling through mTORC1 which renders the inflammatory DCs for long-term commitment towards aerobic glycolysis and anabolic metabolism [75, 76]. In contrast, the TLR-induced long-term glycolytic changes are potentially associated with the mTOR-HIF-*α* axis, via iNOS expression which shuts off electron transport chain (ETC) by nitric oxide (NO) [44, 46, 77]. Taken together, these studies imply the two important metabolic states that exist during DC activation. The early TLR-driven, NO-independent rapid changes in glycolysis are mediated via AKT-driven activation of key glycolytic enzyme HK-II. In aggregate, the long-term NO-dependent commitment of glycolysis is supported by mTORC1 and induction of HIF1*α* (Figure 1).

## 5. Lung DC Subsets and Airway Inflammation

Distinct surface marker expressions and functional properties of DCs have enabled researchers to identify and define different DC subsets that play a crucial role in promoting Th2 immune responses in allergic asthma [6, 7, 78]. Committed DC precursor develops from CDPs in the BM and expresses the hematopoietic cytokine receptor Flt3. CDPs generate pre-cDCs which differentiated into lineage-specific cDC1 and cDC2 and circulate in the bloodstream and lung tissues [4]. Based on their distinct biological functions and surface expressions of integrin molecules, two major lung DC subsets, CD103^+^ DCs (cDC1) and CD11b^+^ (cDC2) DCs, have recently been recognized to play pivotal functions in allergic immune responses [6, 7, 79–81]. Although, CD103^+^ DCs (cDC1) are primarily involved with viral antigens to cross-present CD8^+^ T cells, however, it has recently been showed that in response to an innocuous inhaled antigen house dust mite (HDM) this subset of DCs is particularly capable to prime and mount Th2 immune response [82, 83]. Interestingly, the CD11b^+^ lung DCs (cDC2) are more efficient to trap soluble antigens and to present to CD4^+^ T cells, thereby initiating and producing Th2 immune response in allergic airway inflammations. The CD11b^+^ DC subset in the airway pool is also contributed by circulating monocytes in steady state and also during inflammation, which originates as Ly6C^hi^CCR2^hi^ monocytes from common monocyte progenitors (cMoP) in the BM [19, 79]. These monocyte-derived DCs (moDCs) are primary responders to dose-dependent HDM-induced TLR4 activation and hence migrate to draining the mediastinal lymph node (MLN) to mount Th2 immune response [7]. In contrast, the pDC subset in the lung is apparently involved to balance airway inflammation through interactions with the regulatory T cells (T_regs_).

## 6. Targeting Metabolic Sensors: Implications in Allergic Airway Disease

Allergic asthma is a Th2 disorder of the lung and is manifested by elevated airway inflammation, mucous cell metaplasia with mucous overproductions, airway hyperresponsiveness, and airway remodeling. Airways in severe asthmatics are characterized by increased degree of eosinophilia and elevated numbers of effector CD4^+^ cells in bronchoalveolar lavage that produce canonical set of Th2 cytokines, such as IL4, IL5, and IL13 [84, 85]. The hallmark features of allergic “type-2 high” asthma phenotype in humans are closely manifested in several experimental murine models of allergic asthma, such as administration of either the aeroallergen house dust mite (HDM) or model antigen ovalbumin (OVA), and have been extensively reviewed elsewhere [84, 86, 87]. Here, we describe the current understanding of targeting the important metabolic sensors and signaling pathways to manipulate the adaptive immune responses in allergic asthma (Table 2).

Mouse model-based studies of targeting serine/threonine kinase mammalian target of rapamycin (mTOR) have provided several key insights into the role of mTOR-dependent signaling pathway in the pathogenesis of allergic asthma. Studies using the macrolide product rapamycin (a potent inhibitor of mTOR) in HDM-induced asthma model showed different outcomes in attenuating airway inflammation [88, 89]. For example, surprisingly intranasal administration of HDM with concomitant rapamycin treatment almost significantly attenuated the infiltration of inflammatory cells in particular eosinophils in the BALF and reduction of the Th2 cytokines, such as IL4, IL5, and IL13 levels. The treatment of rapamycin in the induction model also blocked AHR, goblet cell metaplasia, and IgE and activated T cell numbers. However, when rapamycin was treated later following HDM, it failed to reverse and in contrast exacerbated airway inflammation and AHR. These studies confirmed the context-dependent effect of mTOR inhibition by rapamycin treatment and identified the crucial importance of mTOR-mediated signaling pathways in the setting of allergic asthma [88–90].

Moreover, studies using OVA-challenged high-fat diet-induced obese allergic mice have reported that oral treatment with the antihyperglycemic drug (an AMPK activator) metformin significantly abrogates the exacerbation of airway inflammation and lung eosinophilia by NF-*κ*B-dependent iNOS expression [91]. Consistent with this, increased activation of AMPK and reduced oxidative stress were found in metformin-treated allergic mice, whereas, upon allergen challenge, the heterozygous AMPK*α*1-deficient mice showed increased airway inflammation and eosinophil infiltration in the lung [91, 92]. Intriguingly, several studies have identified the importance of the nuclear receptor peroxisome proliferator-activated receptor- (PPAR-) mediated pathways in allergic asthma [65, 93, 94]. Activation of PPARs in DCs by a selective agonist rosiglitazone significantly reduced the migration of DCs from the lung epithelia to the draining lymph nodes and thus inhibited the OVA-specific T cell proliferation [95, 96]. Dendritic cell-specific PPAR activation prevents the Th2-dependent eosinophilic airway inflammation by inducing the anti-inflammatory cytokine IL10 productions. However, the beneficial effect of PPAR activation in mouse asthma models has translated into several discrepancies in the study outcomes from randomized human asthmatic trials. Although in two short-term randomized controlled asthma studies, rosiglitazone has shown to improve the airway inflammation modestly in allergen challenge or smokers with asthma [97, 98]. However, activation of PPARs by pioglitazone failed to improve the mild airway inflammation in obese asthmatics [93, 94]. Collectively, these studies point out the important regulatory role of cooperative immune-metabolic signaling pathways and imply that targeting these metabolic sensors to alter allergic airway inflammation might hold promise as an effective therapeutic strategy for the treatment of asthma. However, future studies warrant more mechanistic research to address the metabolic effects of targeting these metabolic sensors in a cell-specific manner by lung immune cells and structural cells to potentially render adaptive immune response.

## 7. Discussion

Asthma treatment broadly relies on drugs that predominantly aims at relaxing airway smooth muscle (bronchodilators) or used to attenuate airway inflammations (anti-inflammatory drugs). Currently, newer medications, such as leukotriene modifiers along with drug combinations like inhaled corticosteroids with long-acting *β*-adrenergic agonists, are effective as controller therapy. Long-term asthma management with increasing dosage of corticosteroids has potentially become a concern for their adverse effects (refractory-asthma or steroid-unresponsive asthma). Treatments with long-acting *β*-adrenergic agonists, leukotriene modifiers, or anti-IgE therapy suppress airway inflammation and thereby facilitate and reduce the dose and use of inhaled corticosteroids. However, they do not completely cure asthmatic inflammation. Therefore, a comprehensive understanding of how metabolism dictates immune cell fate in DCs are an unmet need to develop novel controller therapeutic options, such as metabolism-based approaches to improve the overall efficacy of asthma management.

A successful airway immune response relies on effective antigen uptake and presentation by DCs to T cells. Targeting metabolic pathways to increase DC function and to modulate immune effector pathways has now come into focus. While inhibition of mTOR pathways in DCs have specific cellular effect on DC maturation and development in cDCs, pDCs, and moDCs [27–29, 99, 100], the administration of mTOR inhibitors in attenuating airway inflammation has varying effects, which abated response in induction model and exacerbation in treatment model [88, 89]. These discrepancies of outcomes are possibly caused by dose, timing, and/or off-target effects. Thus, it is clear that modulating DC-specific mTOR signaling pathways possesses extremely potent immune conditioning effects. In addition to therapies aimed at mTOR, in therapies aimed at other metabolic sensors, such as AMPK, PPARs might prove to be effective when attacking the altered metabolic energetics of DCs [28, 58, 62, 95].

## 8. Conclusions

Emerging immune pathways in asthma pathogenesis have added a new dimension to our understanding of the unique adaptations that contribute to initiate and propagate the adaptive immune responses in airway inflammation. There is a growing appreciation that in order to be effective for antigen uptake, migration to draining lymph node, and antigen presentations, DCs must undergo the correct metabolic reprogramming to initiate appropriate T cell-mediated immune response. Although little research has focused on manipulating DC metabolism to promote activation and priming capacity, evidence is accumulating which addresses how the metabolic perturbations are interlinked to the fundamental properties of DC function and thereby facilitate immune responses. Therefore, integrating DC metabolism in immune therapy design for asthma and airway inflammation promises a novel strategy and can be used to alter the allergen-induced adaptive immune responses.

## Figures and Tables

**Table 1:** Metabolic changes associated with dendritic cell maturation and effector functions^∗^.

References	Cell types	Activation signals	Molecular effect	Metabolic shifts	Immune response
Bajwa et al. [101]	Plasmacytoid DCs (pDCs)	Influenza (flu) and respiratory virus	HIF1*α* translocation (↑)	Glycolysis (↑)	Interferon alpha (IFN-*α*) production and antiviral activity
Wu et al. [102]	Plasmacytoid DCs (pDCs)	TLR-9 agonist (CpG)	PPAR-*α*-dependent FAO activity (↑)	OXPHOS (↑)	Interferon alpha (IFN-*α*) production and antiviral activity
Pantel et al. [103]	CD11C^+^MHC II^+^DX5^−^B220^−^ splenic DCs	TLR-3 agonist (poly I:C)	HIF1*α* expression (↑)	Glycolysis (↑)	Interferon *α*/*β* receptor- (IFNAR-) mediated DC maturation
Malinarich et al. [104]	Monocyte-derived DCs (moDCs)	TLR-4 agonist (LPS)	FAO activity (↑) ATP (↓)	OXPHOS (↑) Glycolytic capacity (↑)	Induce tolerance, negatively regulates immunogenicity
Everts et al. [54]	GM-DCs	TLR-4 agonist (LPS)	HK-II activation	Glycolysis (↑) (early events: within minutes of stimulation)	Activation and function of DC
Everts et al. [75] Amiel et al. [105]	Monocyte-derived DCs (moDCs)	TLR-4 agonist (LPS)	iNOS (↑) mTOR-dependent NO (↓) production	OXPHOS (↓) Glycolysis (↑) (late events: after 24 hours of stimulation)	DC survival and inflammation
Boukhaled et al. [106]	DCs constitutively expressing transcriptional repressor: PCGF6	TLR agonist (LPS)	Active transcriptional silencing by histone demethylation	Glycolysis (↓)	Maintenance of DC quiescence
Wang et al. [30]	Tsc1-deficient bone marrow-derived DCs	Spontaneous	mTORC-1 activity (↓)	Glycolysis (↑) OXPHOS (↑)	Checkpoint of DC development and differentiation; DC-mediated T cell response

^∗^Note that this list is not exhaustive. DC: dendritic cell; HIF1*α*: hypoxia-inducible factor 1*α*; PPAR-*α*: peroxisome proliferator-activated receptor-*α*; FAO: fatty acid oxidase; TLR: toll-like receptor; HK-II: hexokinase II; PCGF6: polycomb group factor 6; Tsc1: tuberous sclerosis 1; ROS: reactive oxygen species; iNOS: inducible nitric oxide synthase; ATP: adenosine triphosphate; mTORC 1: mammalian target of rapamycin complex I.

**Table 2:** Immunological effects of targeting important metabolic signaling pathways in asthma^∗^.

Metabolic sensors/target	Asthma model	Examples of agents/treatments	Asthma phenotype	References
mTOR	OVA-induced	mTORC inhibitor rapamycin derivative (SAR 943)	(↓) airway inflammation, Th2 cytokine production, mucous cell metaplasia, and AHR (↓) CD4^+^ T cell numbers, however, failed to decrease AHR and eosinophilia	Fujitani et al. [90] Eynott et al. [107]
mTOR	Induction and treatment-model of HDM-mediated asthma	mTORC inhibitor Rapamycin	Airway inflammation, Th2 cytokine production, mucous cell metaplasia, and AHR (↓) in induction model, however, exacerbated AHR and airway inflammation when rapamycin administered later in a treatment model	Fredriksson et al. [88] Mushaben et al. [89]
Rheb1	OVA-induced	Myeloid-specific Rheb1 deletion	(↑) eosinophilic airway inflammation, mucous production, and AHR	Kai et al. [108]
AMPK	Obese asthma model: high fat-fed diet + OVA	AMPK activator metformin	(↓) BAL eosinophil counts and iNOS expression in lung	Calixto et al. [91]
AMPK/PPAR-*γ*	OVA-induced	SRT1720; synthetic SRT 1 activator	(↓) BAL eosinophil counts and type 2 cytokine productions	Ichikawa et al. [109]
PPAR-*γ*	FITC-OVA	PPAR-γ agonist rosiglitazone	Inhibits the migration of DCs from airway mucosa to draining lymph nodes and decrease priming of T cells	Angeli et al. [95]
PPAR-*γ*	OVA-pulsed DCs	PPAR-*γ* agonist rosiglitazone	(↓) BAL eosinophil counts and OVA-specific T cell proliferation (↑) interleukin 10 (IL10) production by T cells	Hammad et al. [96]
PPAR-*γ*	Obese asthmatics model of a two-center, 12-week randomized double-blinded trial	PPAR-*γ* agonist pioglitazone	No significant difference in asthma control and treatment group in lung function, (↑) body weight by pioglitazone treatment	Dixon et al. [94]

^∗^Note that this list is not exhaustive. Rheb: Ras homolog enriched in the brain (small GTPase downstream target of tuberous sclerosis complex (TSC) 1/2 and upstream activator of mTORC1); OVA: ovalbumin; HDM: house dust mite; mTORC 1: mammalian target of rapamycin complex I; AMPK: AMP-activated protein kinase; PPAR-*α*: peroxisome proliferator-activated receptor-*α*; BAL: bronchoalveolar lavage; AHR: airway hyperresponsiveness; iNOS: inducible nitric oxide synthase.
